# Gallic Acid Inhibits Bladder Cancer T24 Cell Progression Through Mitochondrial Dysfunction and PI3K/Akt/NF-κB Signaling Suppression

**DOI:** 10.3389/fphar.2020.01222

**Published:** 2020-08-20

**Authors:** Maolin Zeng, Yang Su, Kuangyu Li, Dan Jin, Qiaoling Li, Yan Li, Benhong Zhou

**Affiliations:** ^1^ Department of Pharmacy, Renmin Hospital of Wuhan University, Wuhan, China; ^2^ Department of Pharmacy, Yongchuan Hospital of Chongqing Medical University, Yongchuan, China; ^3^ Department of Urology, The First Affiliated Hospital of Anhui Medical University, Hefei, China; ^4^ Anhui Province Key Laboratory of Genitourinary Diseases, Anhui Medical University, Hefei, China; ^5^ The Institute of Urology, Anhui Medical University, Hefei, China; ^6^ School of Pharmaceutical Sciences, Wuhan University, Wuhan, China; ^7^ Department of Pharmacy, Hubei No.3 People's Hospital of Jianghan University, Wuhan, China

**Keywords:** gallic acid, bladder cancer, proliferation, metastasis, apoptosis

## Abstract

Gallic acid (GA), a hydrolyzable tannin, has a wide range of pharmacological activities. This study revealed that, GA significantly inhibited T24 cells viability in a concentration- and time- dependent manner. The IC_50_ of GA stimulating T24 cells for 24, 48, and 72 h were 21.73, 18.62, and 11.59 µg/ml respectively, and the inhibition rate was significantly higher than the positive control drug selected for CCK-8 assay. Meanwhile, after GA treatment, the morphology of T24 cells were changed significantly. Moreover, GA significantly inhibited T24 cells proliferation and blocked T24 cells cycle in S phase (*p* < 0.001). GA induced T24 cells apoptosis (*p* < 0.001), accompanied by reactive oxygen species (ROS) accumulation and mitochondrial membrane potential (MMP) depolarization. Western blotting analysis showed that GA significantly increased Cleaved caspase-3, Bax, P53, and Cytochrome C (Cyt-c) proteins expression, and decreased Bcl-2, P-PI3K, P-Akt, P-IκBα, P-IKKα, and P-NF-κB p65 proteins expression in T24 cells (*p* < 0.05). Real-Time PCR results verified that GA significantly promoted Caspase-3, Bax, P53, and Cyt-c genes expression, and inhibited Bcl-2, PI3K, Akt, and NF-κB p65 genes expression (*p* < 0.001). However, on the basis of GA (IC_50_) stimulation, NAC (an oxidative stress inhibitor) pretreatment reversed the apoptotic rate of T24 cells and the expression of Bax, Cleaved caspase-3, P53, Bcl-2 proteins, and the MMP level in T24 cells, as well as the expression of Cyt-c protein in T24 cells mitochondria and cytoplasm. In addition, GA significantly suppressed T24 cells migration and invasion ability with VEGF protein inhibition (*p* < 0.001). Briefly, GA can inhibit T24 cells proliferation, metastasis and promote apoptosis, and the pro-apoptotic activity is closely associated with mitochondrial dysfunction and PI3K/Akt/NF-κB signaling suppression. Our study will help in finding a safe and effective treatment for bladder cancer.

## Introduction

Bladder cancer is a common malignant tumor in the urogenital system. It was estimated that 430,000 new cases of bladder cancer were diagnosed worldwide in 2012 (Antoni et al., 2017). Clinically, bladder cancer patients are usually treated with the tumor resection, but even so, the recurrence rate is still very high (Li et al., 2015). Intravesical instillation chemotherapy is needed to reduce the recurrence rate, but these chemotherapy drugs have serious complications, such as urinary pain, hematuria, allergy, and other toxic side effects (Godwin et al., 2018; Harraz et al., 2019). Therefore, it is necessary to find a safe and effective drug to inhibit bladder cancer progression with less adverse effects.

Tannin is rich in nuts, fruits, and other foods, and is a common ingredient in the daily diet. More recent studies have displayed that tannin not only had antioxidant activity, but also showed perfect anti-tumor activity with low toxicity (Anantharaju et al., 2016; Li et al., 2018). Our team have also revealed that pomegranate peel tannins had good anti-bladder cancer activity, and one of the main monomer is gallic acid (GA). GA is a natural polyphenolic compound, also known as 3,4,5-trihydroxybenzoic acid, which belongs to hydrolyzable tannin and widely exists in plants, such as *Rheum rhaponticum* L, *Punica granatum* L, *Rubus idaeus* L, and so on. At present, more and more scholars have paid close attention to GA, due to its extensive pharmacological activities (Karimi-Khouzani et al., 2017; Tsai et al., 2018), simple and clear structure, low price, easy to access, and other advantages. In addition, an increasing number of literature have confirmed GA presented potent pro-apoptotic activity on many types of cancers (Ho et al., 2010; Subramanian et al., 2015; Wang et al., 2016; Lin and Chen, 2017; Gu et al., 2018; Tsai et al., 2018).

As we all know, apoptosis is closely associated with a variety of genes and proteins expression, of which mitochondria plays an important role. Mitochondria is not only a sensor of endogenous apoptosis pathway, but also an amplifier of apoptotic signal, making apoptosis proceeding rapidly and efficiently (Burke, 2017). As apoptosis is initiated, mitochondria activates downstream apoptotic pathway by releasing Cytochrome C (Cyt-c), and then further activating cysteine-aspartic proteases (caspase) (Min et al., 2018). The activated caspase will directly cause intracellular proteins degradation and cytoplasmic nucleus substrates decomposition, and eventually leading to apoptosis (Wen et al., 2019). Several studies have indicated that certain drugs caused cancer cells apoptosis were closely associated with mitochondrial pathway (Song et al., 2016; Wang et al., 2016; Fan et al., 2019). In the latest research, Lin et al. (Lin and Chen, 2017) found that GA could promote human oral cancer SCC-4 cells apoptosis, and the mechanism is related to BIK mediated ROS-dependent apoptotic activity of ER-associated BAX/BAK with Casein Kinase II activation. NF-κB is a transcription factor, and closely associated with genes transcription in immune response and anti-apoptotic aspects (Xu et al., 2018). In addition, Akt, activated by phosphoinositide-3-kinase (PI3K), can participate in different stages of apoptosis by regulating the expression of downstream target proteins such as Bad, Caspase-9, NF-κB, GSK-3, FKHR, p21Cip1, and p27Ki or regulating mitochondria function (Lien et al., 2017; Liu et al., 2018). Madrid et al. (2000) verified that Akt could inhibit apoptosis through activating NF-κB p65. Therefore, PI3K/Akt/NF-κB signaling pathway is very important in cell proliferation and tumor progression, and its inhibition may affect cancer cells proliferation and viability (Ni and Yi, 2017; Yu et al., 2017; Pei et al., 2019). Gu et al. (2018) found that GA significantly induced apoptosis of acute myeloid leukemiacell lines (AML), primary mononuclear cells (MNC) and CD34 stem/progenitors isolated form AML patients *via* Akt/mTOR dependent mitochondrial respiration inhibition, and mitochondrial respiratory inhibition was the result of Akt/mTOR signal inhibition. In addition, Yeh et al. (2008) collected 90 specimens of bladder cancer patients and treated them with NF-κB immunological staining, and the results revealed that nuclear NF-κB can be served as an important predictor of specific and overall survival rate of bladder cancer patients, so it is expected to be a therapeutic target for bladder cancer.

Although some studies have indicated GA has significant anti-tumor activity, the specific mechanism of its pro-apoptosis activity is not yet well-elucidated. Considering this situation, the purpose of our research is to explore the effects of GA on bladder cancer progression. We used human bladder cancer T24 cell as the target cell, and assessed the effects of GA on T24 cells proliferation, apoptosis, cell cycle distribution, and metastasis. In addition, we explored the involved mechanism and determined whether the apoptosis induced by GA is associated with mitochondrial dysfunction and PI3K/Akt/NF-κB signaling inhibition.

## Materials and Methods

### Reagents

GA with 99% purity, 5-Fluorouracil (5-FU) and N-acetylcysteine (NAC) were obtained from Sigma (USA). Punicalagin, ellagic acid and punicalin with 99% purity were purchased from Chengdu Institute of biology, Chinese Academy of Sciences (Chengdu, China). CCK-8 kit was acquired from Dojindo (Japan). The 5-ethynyl-2-deoxyuridine (EdU) labeling/detection kit was purchased from Ribobio (Guangzhou, China). Annexin V-FITC apoptosis detection kit and PI/RNase Staining Buffer kit were obtained from BD Biosciences (Becton Dickinson, USA). ROS detection kit was obtained from Jiancheng Bioengineering (Nanjing, China). Mitochondrial Membrane Potential (MMP) detection kit was obtained from BestBio company (Shanghai, China). The primary antibodies against Cleaved Caspase-3, Caspase-3, P53, Bcl-2, Bax, Cyt-c, P-Akt, Akt, P-NF-κB p65, NF-κB p65, and P-IκBα were purchased from Cell Signaling Technology (CST, USA). The primary antibodies against PI3K, P-PI3K, P-IKKα, VEGF, COX-IV, and β-Actin were purchased from abcam (UK). The second antibody was obtained from LI-COR (USA). PrimeScipt™ RT reagent Kit with gDNA Eraser and SYBR@Premix Ex Taq™ II were obtained from Takara Bio (Japan). Minimum essential medium (MEM), fetal bovine serum (FBS), penicillin-streptomycin were purchased from Gibco (Grand Island, USA). Matrigel was obtained from BD Biosciences (San Jose, USA).

### Cell Culture and Drug Preparation

The human bladder cancer T24 cell line was obtained from China Center for Type Culture Collection (CCTCC, No : GDC078). T24 cells were cultured in MEM medium (Gibco) with 10% FBS, 100 units/ml penicillin and 100 units/ml streptomycin, then maintained in 37°C with 5% CO_2_ incubator (Binder, Germany). Preparing GA stock solution at a concentration of 500 µg/ml: precisely weighed 2.00 mg GA in a EP tube, added 4 ml FBS free MEM medium, mixed with a vortex to ensure complete dissolution, and then stored at -20°C. The GA stock solution needs to be diluted before use. Specifically, the GA stock solution was taken out and dissolved in the dark at 37°C, and then it was diluted to the appointed concentration with FBS-free MEM medium to obtain the working solution. Attention should be paid here to avoid light in the use of GA.

### Cell Viability Assay

For cell counting kit-8 (CCK-8) assay, T24 cells were firstly plated in a 96-well plate for 24 h incubation. After that, the previous medium was removed, and 100 µl 0, 6.25, 12.5, 25, 50, 75, and 100 µg/ml GA or 5-FU solution were respectively added into each well for 24, 48, and 72 h stimulation. After reaching the appointed time, the previous medium was replaced with 100 µl new MEM medium containing 5% CCK-8. After 1 h incubation at 37°C in the dark, the absorbance at 450 nm was measured by a microplate reader (Perkin Elmer, USA). Finally, the inhibition rate and half inhibition concentration (IC_50_) in each group were calculated.

### Cellular Morphology Analysis

T24 cells were firstly plated in a 6-well plate for 48 h incubation. After that, the previous medium was removed, and 100 µl different concentration of GA (0, 6.25, 12.5, and 25 µg/ml) were added into each well. After 24 h stimulation, the cell morphological changes in each group were observed by an inverted microscope (Olympus, Japan).

### Plate Clone Formation Assay

T24 cells were firstly plated in a 6-well plate and cultured for 48 h, the cells were then treated with different concentrations of GA (0, 6.25, 12.5, 25 µg/ml) for 24 h. Subsequently, the cells in each well were digested, counted, and plated into a new 6-well plate with complete media at a density of 2×10^3^ cells/well, and consecutively cultured for one week at 37°C. After the medium was removed, the cells were washed with phosphate buffered saline (PBS) for 3 times. After being fixed with 4% paraformaldehyde for 15 min, the cells were stained with 0.5% crystal violet solution for 15 min. Finally, the cells were washed with PBS and dried in the air, and the number of colonies was counted manually.

### Ethynyldeoxyuridine (EdU) Staining

T24 cells were firstly plated in a 6-well plate with equal volume and cultured for 48 h at 37°C with 5% CO_2_. The cells were then treated with different concentrations of GA (0, 6.25, 12.5, 25 µg/ml) for 24 h. Subsequently, the EdU working solution (20 µM) was added to each well with equal volume and incubated for 2 h. After treatment with 4% paraformaldehyde and PBS containing 0.3% Triton X-100, the cells were stained with the EdU reaction liquid according to the instructions of 5-ethynyl-2-deoxyuridine labeling/detection kit (Ribobio, Guangzhou, China). DAPI was used to label the nuclei for 15 min after PBS washing. Finally, the EdU-positive cells in five randomly selected fields were viewed by a fluorescence microscopy (Olympus, Japan).

### Cell Cycle Analysis

T24 cells were firstly stimulated with an increasing concentration of GA solution (0, 6.25, 12.5, 25 µg/ml) for 24 h. After that, the cells were collected and washed with PBS. 1 ml 70% ethanol was added into each tube and incubated at -20°C overnight for fixing. Subsequently, the fixed T24 cells were collected and washed with 4°C PBS, and 500 µl PI/RNase dye solution was added into each tube and incubated for 15 min at 25°C in the dark. Finally, the fluorescence intensity of each group was detected immediately using the flow cytometry (BD Biosciences, USA).

### Cell Apoptosis Analysis

Briefly, T24 cells were firstly stimulated with an increasing concentration of GA solution (0, 6.25, 12.5, 25 µg/ml). After 24 h treatment, the cells were collected by centrifugation after trypsinization, and washed with cold PBS. Then 500 µl binding buffer solution was added into each tube for resuspension. Finally, 5 µl fluorescein-conjugated Annexin V solution and 5 µl PI solution were added into each tube respectively and incubated for 15 min at 25°C in the dark. Finally, the apoptosis rates of T24 cells were detected using the flow cytometry (BD Biosciences, USA).

### Intracellular ROS Production

T24 cells were firstly stimulated with an increasing concentration of GA solution (0, 6.25, 12.5, 25 µg/ml) for 24 h. After stimulation, T24 cells were digested by trypsin and collected by centrifugation. Then 1 ml DCFH-DA working solution was added to each tube and dyed for 30 min at 25°C in the dark. After incubation, the cells were obtained and washed with PBS to remove the excess DCFH-DA. Finally, the fluorescence intensity was immediately detected by a microplate reader (Perkin Elmer, USA) after adjusting the consistency of cell density.

### MMP Level Assessment

The JC-1 staining method was used to measure the changes in T24 cells MMP level after GA stimulation. Briefly, T24 cells were firstly stimulated with an increasing concentration of GA solution (0, 6.25, 12.5, 25 µg/ml) for 24 h. After stimulation, the cells were collected and washed with PBS. The JC-1 working solution was added to each tube and dyed for 15 min at 25°C in the dark. Then the cells were obtained by centrifugation and resuspended with PBS. Finally, the MMP level changes were detected using the flow cytometry (BD Biosciences, USA). In addition, the evaluation of MMP level was also performed with a fluorogenic lipophilic cation, according to the manufacturer’s protocol. In cells with hyper-polarized mitochondrial membranes, JC-1 spontaneously forms complexes (J-aggregates) emitting red fluorescence. In cells with depolarized mitochondrial membranes, JC-1 remains in the monomeric form, emitting green fluorescence. The detection of MMP was performed by fluorescent microscopy (200×magnification). All experiments were repeated three times.

### Western Blotting Analysis

T24 cells were firstly plated in a 6-well plate for 48 h incubation. After that, the previous medium in each well was removed and replaced with different concentrations of GA solution (0, 6.25, 12.5, 25 µg/ml). After 24 h stimulation, the 6-well plate was moved on the ice and washed by PBS. Then the cells in each well were incubated with 600 µl RIPA lysis buffer solution, and lysed on ice for 20 min, and scraped with a cell scraper, then transferred into an EP tube. The total protein was obtained by collecting supernatant after centrifugation. The BCA method was used to detect the extracted protein concentration, and the sample volume was adjusted according to the protein concentration before electrophoresis. Then the sample was added into each pore respectively and the marker was added at both ends, 10% Tris–Glycine gels were used to separate the protein sample under constant current. After that, the protein gelatin was transferred to PVDF membranes. Five percent BSA in TBST (TBS with 0.1% Tween-20) was used to block the membranes for 1 h at room temperature. Then the membranes were incubated with the specific primary antibodies against Bax, Bcl-2, Caspase-3, Cyt-c, P53, P-Akt, Akt, P-PI3K, PI3K, P-IκBα, P-IKKα, P-NF-κB p65, NF-κB p65, VEGF, COX-IV, and β-Actin at 4°C overnight respectively. The next day, the membranes were washed with TBST for three times, and incubated with the corresponding secondary antibody (LI-COR, USA) for 1 h at room temperature in the dark. The membranes were scanned by Odyssey imaging system (USA). Finally, the relative gray value of the target proteins and internal reference protein were respectively measured by Image J Software.

### Quantitative Real-Time Polymerase Chain Reaction (QRT-PCR) Analysis

In our experiment, the expression of the target genes was regarded as the mRNA level. The relative expression of Bax, Bcl-2, Cyt-c, Caspase-3, P53, PI3K, Akt, and NF-κB p65 genes in T24 cells after GA stimulation were detected by RT-PCR method. In short, T24 cells were firstly stimulated with an increasing concentration of GA solution respectively. After 24 h stimulation, the cells were collected through trypsinization and transferred to the EP tubes. Trizol was then added into each tube to extract the total RNA according to the manufacturer’s protocol. The ultraviolet spectrophotometer (Beckman, USA) was used to detect the concentration and purity of the extracted RNA from each group. The reverse transcription was performed using an ABI PCR instrument (Thermo Fisher, USA) with GAPDH as the internal reference. The primer sequence was obtained from Wuhan Google Biotechnology. The design and synthesis of the primer sequences were shown in **Table 1**. Finally, the genes mRNA relative expression was detected by an AB7500 detection system (Thermo Fisher, USA).

**Table 1:** Sequences of Real-Time Polymerase Chain Reaction (RT- PCR) Primer.

	Forward primer 5’ to 3’	Reverse primer 5’ to 3’
Bax	AAGAAGCTGAGCGAGTGTCT	GTTCTGATCAGTTCCGGCAC
Bcl-2	GCCTTCTTTGAGTTCGGTGG	GAAATCAAACAGAGGCCGCA
Caspase-3	ACTGGACTGTGGCATTGAGA	GCACAAAGCGACTGGATGAA
Cyt-c	GGGTGATGTTGAGAAAGGCA	TCCCCAGATGATGCCTTTGT
P53	GTCCAGATGAAGCTCCCAGA	CAAGGCCTCATTCAGCTCTC
PI3K	GTCCTATTGTCGTGCATGTGG	TGGGTTCTCCCAATTCAACC
AKT	TTCTATGGCGCTGAGATTGTGT	GCCGTAGTCATTGTCCTCCAG
NF-κB p65	CACCGGATTGAGGAGAAACG	GGGAAGGCACAGCAATGC
GAPDH	TCAAGAAGGTGGTGAAGCAGG	TCAAAGGTGGAGGAGTGGGT

### Wound Healing Assay

T24 cells from each group were plated in 6-well plates for 48–72 h. When the cell density reached approximately 80%, the monolayer was wounded by scratching with a 200 µl sterile pipette tip lengthwise along the plate surface, and the media was removed. The cells were then washed 2–3 times with PBS and cultured in serum-free media. Images of cell migration were captured at 0, 12, and 24 h by an inverted microscope (Olympus, Japan), and the area of migration (µm^2^) was measured with ImageJ software.

### Transwell Migration and Invasion Assay

For transwell migration assay, T24 cells were firstly stimulated with an increasing concentration of GA solution (0, 6.25, 12.5, 25 µg/ml). After 24 h stimulation, the cells were collected by trypsin and suspended in FBS-free MEM medium. After adjusting the density of cells, we added 100 µl 1×10^5^ cells/ml T24 cells suspension on the upper boyden chamber (Corning, USA), and added 700 µl MEM medium containing 20% FBS on the lower chamber, and placed the 24-well plate in 37°C incubator. After 24 h incubation, the transwell chamber was taken out from the incubator. The non-migrated cells were scraped with a cotton swab. Then the migrated cells were fixed with 95% methanol for 15 min, and then dyed with 0.5% crystal violet solution for 15 min. Finally, the migrated cells were observed and photographed by an inverted microscope (Olympus, Japan). The cells in 5 random fields were counted and the mean was calculated. For transwell invasion assay, the matrigel was firstly diluted in a ratio of 1:8 with FBS-free MEM medium. The transwell upper chambers were then coated with 80 µl diluted matrigel. The subsequent operations were the same as the migration experiments.

### Enzyme-Linked Immunosorbent Assay (ELISA) Analysis

The samples were collected from T24 cells culture supernatant and centrifuged at 4°C, 2,000×g for 10 min. The separated supernatant was used for biochemical analysis. The level of VEGF (cat. no. EK0393) was measured using an ELISA kit purchased from Wuhan Boster Biological Technology, Ltd. (Wuhan, China). The absorbance at 450 nm was measured on a microplate reader (BioTek Elx9808, BioTek Instruments, Inc., Winooski, VT, USA).

### Statistical Analysis

All experiments were repeated at least three times independently. The data were expressed as the mean ± standard deviation (SD). The treated groups were compared by one-way variance (ANOVA) with SPSS 13.0 (IBM, USA). The statistically significant *p* values were labeled as follows: ^*^ *p*<0.05, ^**^p<0.01, ^***^p<0.001.

## Results

### GA Inhibits T24 Cells Viability

**Figure 1:**
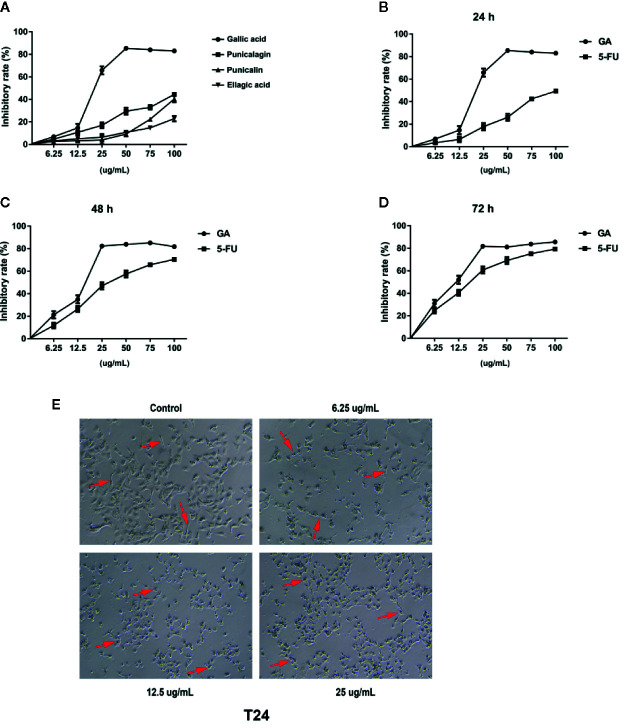
GA inhibited T24 cells viability and affected cells morphology. **(A)** The viability of T24 cells with different concentrations of monomers in pomegranate peel tannins treatment were measured respectively by CCK-8 assay. **(B–D)** The viability of T24 cells with different concentrations of GA treatment in 24, 48, and 72 h were measured by CCK-8 assay. **(E)** The changes of T24 cells morphology with different concentrations of GA treatment were observed by an inverted microscope (40× magnification). The red arrow refers to T24 cell.

In order to find the effective anti-cancer component in pomegranate peel tannins, we firstly compared the effects of each monomer in pomegranate peel tannins on T24 cells viability, and found the inhibition of GA on T24 cells viability was significantly higher than the other three monomers (**Figure 1A**). Subsequently, in order to measure the effects of GA on T24 cells viability, CCK-8 assay was used. Furthermore, 5-fluorouracil (5-FU) was selected as a positive control. The results were represented in **Figures 1B–D**, which revealed that the inhibition of GA on T24 cells viability was significantly higher than 5-FU with the same concentration and time, especially for stimulating with 24 and 48 h (*p* < 0.001). The half inhibitory concentration (IC_50_) of GA stimulating T24 cells for 24, 48, and 72 h were 21.73, 18.62, and 11.59 µg/ml respectively, while the IC_50_ of 5-FU were 102.04, 35.63, and 20.01 µg/ml respectively. Compared with the control group, T24 cells viability in GA-treated group was significantly decreased, indicating that GA significantly inhibited T24 cells viability in a time- and concentration-dependent manner (*p* < 0.01). As GA stimulated T24 cells for 24 h, the inhibition rate of GA with 6.25 and 25 µg/ml was 6.89 and 65.77%, respectively. These results indicated that GA significantly inhibited T24 cells viability. Combining with the results of CCK-8, we determined GA with 6.25, 12.5, and 25 µg/ml as the appropriate action concentration and 24 h as the appropriate action time.

### GA Changes T24 Cells Morphology

In order to observe the effects of GA on T24 cells morphology, we used the inverted microscope, and found that T24 cells were elongated, with strong adherence, large cell bodies, clear cell contour, and cells were in a good condition. After stimulating with GA for 24 h, T24 cells were shrank, with weak adherence, loose intercellular contact, blurred cell contour, and cells were in an apoptotic state (**Figure 1E**).

### GA Inhibits T24 Cells Proliferation

**Figure 2:**
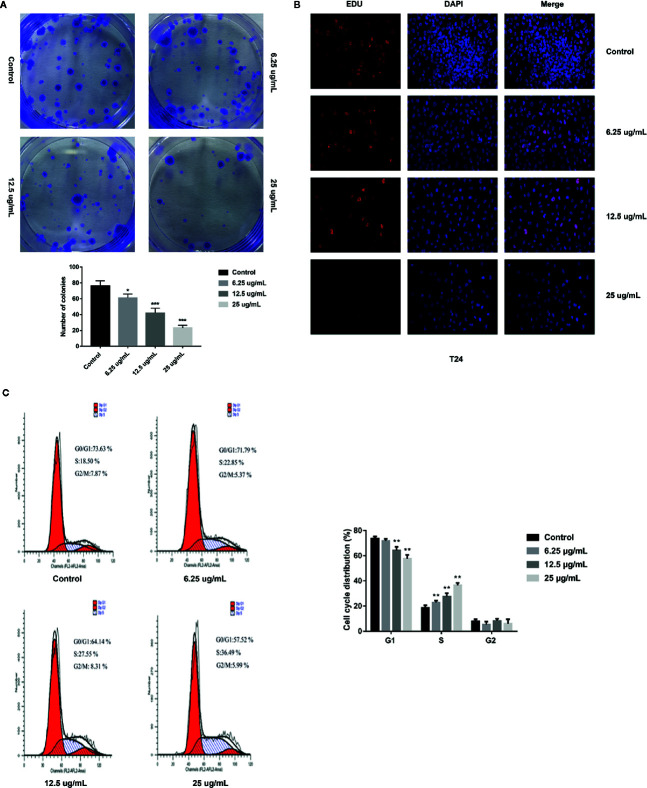
GA affected T24 cells proliferation and cells cycle distribution. **(A)** Representative images and quantitative analyses of T24 cell colonies in each group were displayed according to the plate cloning assays. **(B)** Representative fluorescent images of proliferating T24 cell nuclei were shown by EdU staining (200× magnification). **(C)** Representative images and quantitative analyses of T24 cell cycle status were assessed by flow cytometry. ^*^ *p* < 0.05, ^**^ *p* < 0.01, ^***^ *p* < 0.001, compared to the control group.

In order to further assess the effects of GA on T24 cells proliferation, we adopted the plate clone formation assay and EdU staining. The plate cloning assay results showed that there were significantly fewer number of colonies of T24 cells in the GA-treated groups than in the control group, and the number of colonies were decreasing with an increasing concentrations of GA (**Figure 2A**). The EdU staining results showed that there were significantly fewer fluorescent dots indicating nuclei in T24 cells in the GA-treated groups than in the control group, and the number of fluorescent dots were decreasing with an increasing concentrations of GA (**Figure 2B**). These results indicated that GA suppressed T24 cells proliferation in a concentration dependent way.

### GA Affects T24 Cells Cycle Distribution

In order to assess the effects of GA on T24 cells cycle, we adopted the flow cytometry, and found that, in the control group, the percentage of T24 cells in G1 phase was 73.63%, while that in S phase was 18.50%. With the increasing concentration of GA, the number of T24 cells in S phase was significantly increased, while the number of T24 cells in G1 phase was significantly decreased (*p* < 0.01). In the group of GA with 25 µg/ml, the percentage of cells in G1 phase was 57.52%, while that in S phase was 36.49% (**Figure 2C**). These results showed that GA blocked T24 cells cycle in S phase.

### GA Induces T24 Cells Apoptosis

**Figure 3:**
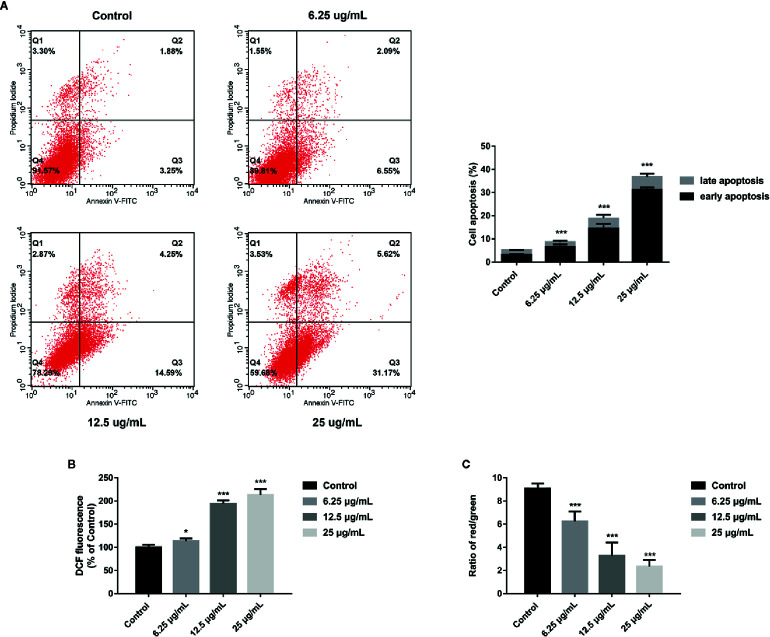
GA induced T24 cells apoptosis and ROS accumulation as well as MMP depolarization. **(A)** Representative images and quantitative analyses of T24 cells apoptosis in each group were measured by flow cytometry. **(B)** Quantitative analyses of ROS level in T24 cells with different concentrations of GA treatment were measured by a microplate reader. **(C)** Quantitative analyses of MMP level in T24 cells with different concentrations of GA were assessed by flow cytometry. ^*^ *p* < 0.05, ^***^ *p* < 0.001, compared to the control group.

In order to explore whether GA could induce T24 cells apoptosis, the FITC/PI staining method was used, and the results were displayed in **Figure 3A**, in which, the right upper quadrant represented the late stage apoptotic cells, and the right lower quadrant represented the early stage apoptotic cells. The results revealed that GA significantly induced T24 cells apoptosis, and the apoptotic rate of T24 cells in GA-treated groups were significantly higher than the control group (*p* < 0.01). The apoptotic rate of T24 cells in the control group was 5.13%, while the apoptotic rate in GA-treated groups with 6.25, 12.5, and 25 µg/ml were 8.64, 18.84, and 36.79% respectively (*p* < 0.01). In addition, the early apoptotic rate was 3.25% in the control group and 31.17% in GA (25 µg/ml) treated group, indicating that GA mainly caused T24 cells early apoptosis. These results suggested that GA promoted T24 cells apoptosis in a concentration-dependent manner, and mainly induced early apoptosis of T24 cells.

### GA Promotes Intracellular ROS Generation

Current researches have revealed that mitochondrial ROS plays a vital role in cell apoptosis, and the mechanism is involved in many aspects of apoptosis (Moloney and Cotter, 2018). Since GA-induced T24 cells apoptosis may be caused by the generation of intracellular ROS, the level of mitochondrial ROS in T24 cells was measured. The DCFH-DA fluorescent probes were used to detect intracellular ROS accumulation to assess the degree of cell damage. The results were shown in **Figure 3B**, after GA stimulation for 24 h, the relative levels of ROS in T24 cells in GA-treated group with 6.25, 12.5, and 25 µg/ml were 113.4, 194.3, and 213.1% respectively. Compared with the control group, the levels of ROS were significantly increased in GA-treated groups (*p* < 0.05). These results indicated that GA induced oxidative stress in T24 cells.

### GA Reduces Intracellular MMP Level

An obvious decrease in MMP level is generally regarded as the earliest change in apoptosis. The MMP level changes in T24 cells were expressed as the ratio of red/green fluorescence after stimulating with GA. As shown in **Figure 3C**, the cells in the control group had strong red fluorescence and weak green fluorescence. After GA stimulation, the red fluorescence was getting weaker and the green fluorescence was getting stronger. The red/green ratio in the control group was 9.08 ± 0.13, while the ratios in GA-treated groups with 6.25, 12.5, and 25 µg/ml were 6.24 ± 1.12, 3.28 ± 1.58, and 2.34 ± 0.91, respectively. Compared with the control group, the MMP levels in GA-treated groups were significantly decreased (*p* < 0.001). These results indicated that GA caused the decline of MMP level in T24 cells, and implied that GA-induced T24 cells apoptosis might be associated with the mitochondrial dysfunction.

### GA Regulates Mitochondrial Dysfunction-Related Apoptosis Markers Expression

**Figure 4:**
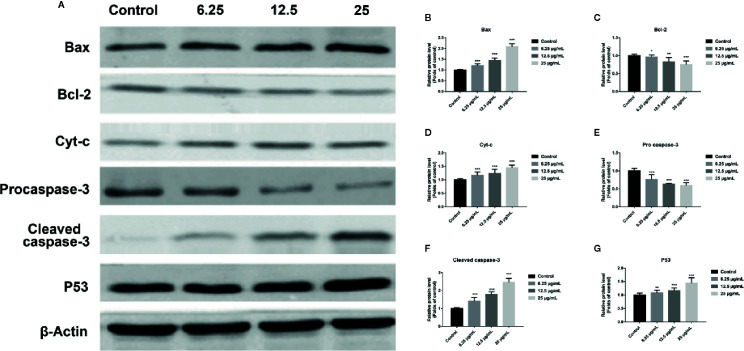
GA affected the expression of mitochondrial dysfunction related apoptosis proteins in T24 cells. **(A–G)** Representative western blot images and quantitative analyses of apoptosis-related proteins, including Bax **(A, B)**, Bcl-2 **(A, C)**, Cyt-c **(A, D)**, Pro-caspase-3 **(A, E)**, Cleaved caspase-3 **(A, F),** and P53 **(A, G)**, in T24 cells with different concentrations of GA treatment. ^*^ *p* < 0.05, ^**^ *p* < 0.01, ^***^ *p* < 0.001, compared to the control group. β-Actin was served as the internal reference.

In order to further assess whether GA could induce T24 cells apoptosis, and verify this process was associated with the mitochondrial dysfunction, we detected the relevant proteins and genes expression. The results showed that, compared with the control group, the expression of Bax protein was significantly up-regulated while the expression of Bcl-2 and Procaspase-3 proteins were significantly down-regulated, and the ratio of Bax/Bcl-2 was significantly increased in GA-treated groups (*p* < 0.001). Meanwhile, the expression of P53, Cyt-c, and Cleaved caspase-3 proteins were significantly increased (**Figure 4**). In addition, compared with the control group, the level of Bcl-2 mRNA was lower in GA-treated group (**Figure 7C**). GA increased the level of Bax mRNA by 1-, 2.18-, 4.84-, and 8.14-fold (**Figure 7A**); Caspase-3 mRNA by 1-, 3.13-, 4.59-, and 6.01-fold (**Figure 7B**); P53 mRNA by 1-, 1.71-, 2.81-, and 3.19-fold (**Figure 7D**); Cyt-c mRNA by 1-, 2.09-, 4.68-, and 5.79-fold (**Figure 7E**), respectively in T24 cells. These results suggested that GA-induced T24 cells apoptosis was associated with the mitochondrial pathway.

### GA Induces T24 cells Apoptosis *via* the Mitochondrial Pathway

**Figure 5:**
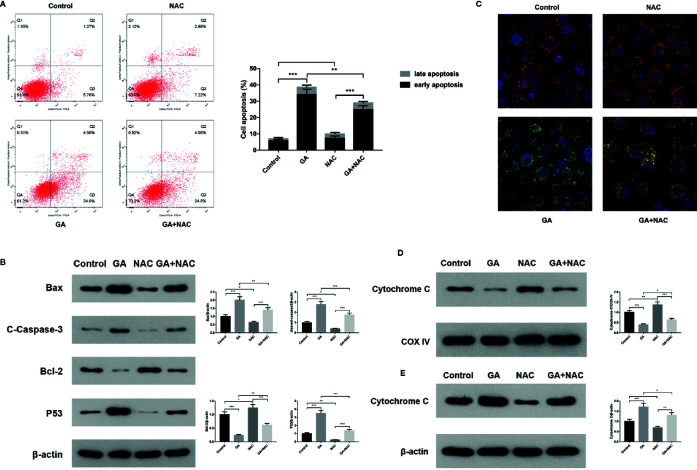
NAC reversed apoptosis of T24 cells induced by GA. **(A)** Representative images and quantitative analyses of T24 cells apoptosis in each group were measured by flow cytometry. **(B)** Representative western blot images and quantitative analyses of mitochondrial dysfunction related apoptosis proteins including Bax, Bcl-2, P53, and Cleaved caspase-3 in T24 cells. **(C)** Representative images of MMP level in T24 cells in each group were measured by the laser scanning confocal microscope. Red indicates normal mitochondria, green indicates depolarized mitochondria, blue indicates the cell nucleus. **(D)** Representative western blot images and quantitative analyses of Cytochrome C expression in T24 cells mitochondria. **(E)** Representative western blot images and quantitative analyses of Cytochrome C expression in T24 cells cytoplasm. ^*^ *p* < 0.05, ^**^ *p* < 0.01, ^***^ *p* < 0.001, compared to the control group and GA (IC_50_) group. β-Actin and COX-IV were served as the internal reference.

In order to further verify T24 cells apoptosis induced by GA was associated with the mitochondrial dysfunction, we used an oxidative stress inhibitor NAC and detected the relevant markers expression. The experiments of this part were divided into four groups, including Control group, NAC group (10 mmol/L NAC pretreatment for 4 h, then replaced with fresh culture media), GA group (IC_50_ for 24 h), NAC + GA group (NAC pretreatment for 4 h, then replaced with GA IC_50_ for 24 h). The results showed that, compared with the control group, the apoptotic rate of T24 cells was significantly increased in GA (IC_50_) group (*p* < 0.01), while compared with the GA (IC_50_) group, the apoptotic rate of T24 cells was significantly decreased in GA + NAC group (**Figure 5A**). Meanwhile, compared with the control group, the expression of Bax, Cleaved caspase-3, and P53 proteins were significantly increased while the expression of Bcl-2 was significantly decreased in GA (IC_50_) group (*p* < 0.01), and NAC reversed this effect (**Figure 5B**). Moreover, compared with the control group, the intensity of green fluorescence in T24 cells was significantly increased and the intensity of red fluorescence in T24 cells was significantly decreased in GA (IC_50_) group (*p* < 0.01), while compared with the GA (IC_50_) group, the intensity of green fluorescence in T24 cells was significantly decreased and the intensity of red fluorescence in T24 cells was significantly increased in GA + NAC group (**Figure 5C**). In addition, compared with the control group, the expression of Cyt-c protein in T24 cells mitochondria was significantly decreased in GA (IC_50_) group (*p* < 0.01), while compared with the GA (IC_50_) group, the expression of Cyt-c protein in T24 cells mitochondria was significantly increased in GA+NAC group (**Figure 5D**). Meanwhile, compared with the control group, the expression of Cyt-c protein in T24 cells cytoplasm was significantly increased in GA (IC_50_) group (*p* < 0.01), while compared with the GA (IC_50_) group, the expression of Cyt-c protein in T24 cells cytoplasm was significantly decreased in GA + NAC group (**Figure 5E**). These results further suggested that GA-induced T24 cells apoptosis was closely associated with the mitochondrial pathway.

### GA Induces T24 Cells Apoptosis *via* the PI3K/Akt/NF-κB Signaling Pathway

**Figure 6:**
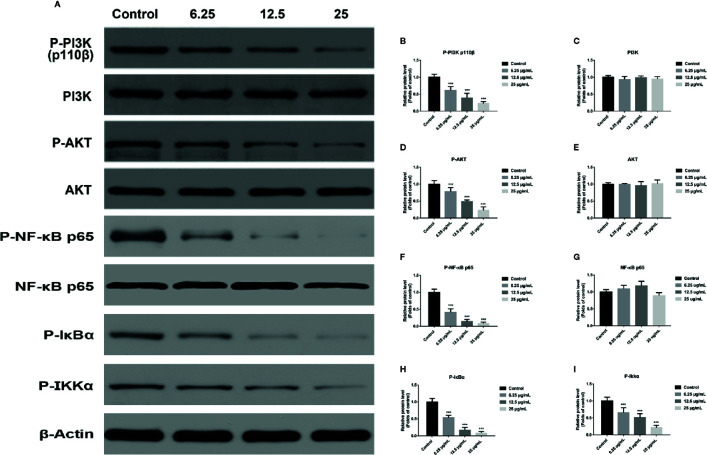
GA induced T24 cell apoptosis *via* the PI3K/Akt/NF-κB signaling pathway. **(A–I)** Representative western blot images and quantitative analyses of several critical proteins, including P-PI3K **(A, B)**, PI3K **(A, C)**, P-Akt **(A, D)**, Akt **(A, E)**, P-NF-κB p65 **(A, F)**, NF-κB p65 **(A, G)**, P-IκBα **(A, H),** and P-IKKα **(A, I)** in T24 cells with different concentrations of GA treatment. ^***^ *p* < 0.001, compared to the control group. β-Actin was served as the internal reference.

**Figure 7:**
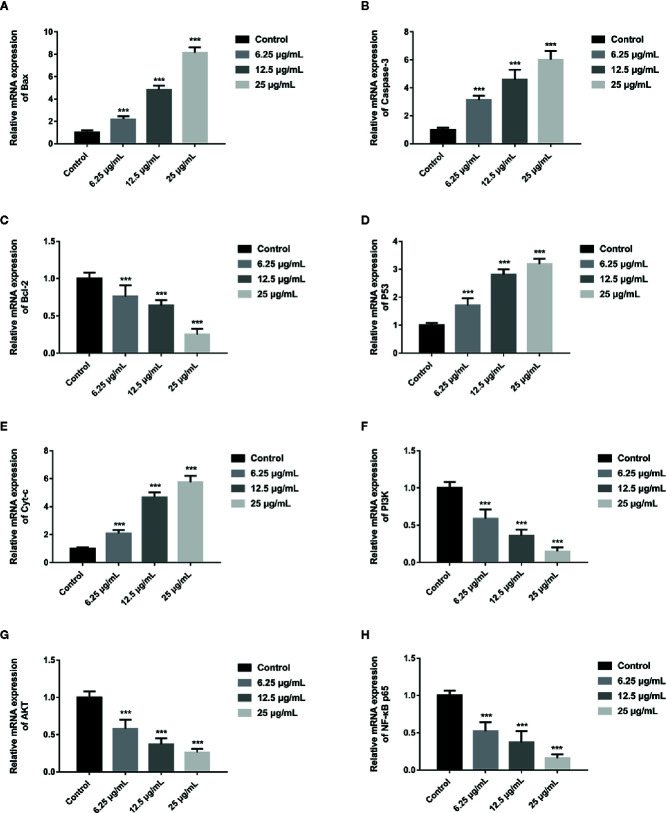
GA affected the relative expression of mitochondrial pathway related apoptosis genes and PI3K/Akt/NF-κB signaling pathway related genes in T24 cells. **(A–H)** Quantitative analyses of several critical genes, including Bax **(A)**, Caspase-3 **(B)**, Bcl-2 **(C)**, P53 **(D)**, Cyt-c **(E)**, PI3K **(F)**, Akt **(G),** and NF-κB p65 **(H)**, in T24 cells with different concentrations of GA treatment. ^***^ *p* < 0.001, compared to the control group.

In order to explore the potential molecular mechanism involved in GA promoting T24 cells apoptosis, we evaluated the relative expression of PI3K, P-PI3K, Akt, P-Akt, NF-κB p65, P-NF-κB p65, P-IκBα, and P-IKKα proteins. We found that after GA stimulation, the expression of P-PI3K and P-Akt proteins in T24 cells were significantly decreased, but there was no significant difference in the expression of PI3K and Akt proteins in T24 cells (**Figure 6**). Furthermore, after GA stimulation, the expression of P-IκBα, P-IKKα, and P-NF-κB p65 proteins in T24 cells were significantly decreased, but there was no significant difference in the expression of NF-κB p65 protein in T24 cells (**Figure 6**). In addition, we evaluated the relative expression of PI3K, Akt, and NF-κB p65 mRNA, and found that the relative mRNA levels of PI3K, Akt, and NF-κB p65 in T24 cells were significantly down-regulated after GA stimulation. As an increasing concentration of GA, the relative levels of PI3K mRNA were 1, 0.59, 0.36, and 0.15 respectively (**Figure 7F**), the relative levels of Akt mRNA were 1, 0.58, 0.37, and 0.26 respectively (**Figure 7G**), and the relative levels of NF-κB p65 mRNA were 1, 0.52, 0.37, and 0.16 respectively (**Figure 7H**). These results indicated that GA induced T24 cells apoptosis *via* inhibiting the PI3K/Akt/NF-κB signaling pathway.

### GA Inhibits T24 Cells Migration and Invasion

**Figure 8:**
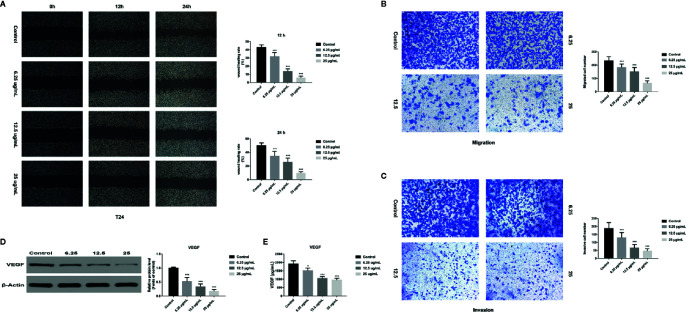
GA affected T24 cell migration and invasion in vitro. **(A)** Representative images and quantitative analyses of T24 cell migration rates in each group were shown according to the scratch assay (200×magnification). **(B)** Representative images and quantitative analyses of penetrated T24 cells in each group were shown according to transwell migration assay (200×magnification). **(C)** Representative images and quantitative analyses of invasive T24 cells in each group were displayed according to transwell invasion assay (200× magnification). **(D)** Representative western blot images and quantitative analyses of VEGF protein in T24 cells under different concentrations of GA treatment. **(E)** Quantitative analyses of VEGF in T24 cells supernatant under different concentrations of GA treatment. ^*^ *p* < 0.05, ^***^ *p* < 0.001, compared to the control group. β-Actin was served as the internal reference.

In order to assess the effects of GA on T24 cells migration and invasion, we used the wound healing assay and transwell assay and found that, compared with that in the control group, the migration rate of T24 cells was significantly inhibited in GA-treated group, and the inhibition rate was GA-concentration dependent (**Figure 8A**). Moreover, we found that the number of T24 cells passed through the chamber membrane was significantly decreased after GA treatment, as shown in **Figure 8B**. For transwell invasion assay, we found that, as the increasing concentrations of GA, the number of T24 cells passed through the matrigel and chamber membrane was getting fewer, as shown in **Figure 8C**. In addition, we used western blotting assay and ELISA analysis to detect the expression of VEGF protein in T24 cells and secretive VEGF from T24 cells after GA stimulation. We found that GA significantly inhibited VEGF protein expression in T24 cells (**Figure 8D**), and GA significantly inhibited VEGF secretion into T24 cells supernatant (**Figure 8E**). These results indicated that GA significantly inhibited the migration and invasion of T24 cells as well as the expression of VEGF protein in T24 cells.

## Discussion

Bladder cancer is one of the most common malignant tumor of urinary system with poor prognosis and low survival rate. As the patients with bladder cancer have clinical symptoms, the course of this disease may have arrived the middle or late stage. In this case, even receiving treatment, it is very easy to relapse (Schulz et al., 2019). Therefore, looking for a safe and effective drug to further improve the bladder cancer patients survival rate is so necessary. Recently, more and more scholars are committed to find the new and effective anticancer drugs from natural products. Our team mainly studied the pharmacological activities of tannins, and found that pomegranate peel tannins can induce bladder cancer T24 cells apoptosis. In addition, we detected the main monomers in pomegranate peel tannins by HPLC-ESI-MS method, and found the main monomers were punicalagin, punicalin, ellagic acid, and GA. Therefore, on the basis of our previous research, we firstly compared the four monomers and selected the most effective one on inhibiting T24 cells proliferation. After stimulating with these monomers for 24 h respectively, the inhibition rates were measured by CCK-8 assay. The results revealed that GA possessed the strongest inhibitory effect on T24 cells viability, followed by punicalagin, punicalin, and ellagic acid (**Figure 1A**). In order to further explore the inhibitory effect of GA on T24 cells viability, we selected 5-FU as the positive control. 5-FU is a common anti-neoplastic drug that can interfere DNA synthesis mainly acting on S phase of cell cycle, and it is also a chemotherapy drug for bladder irrigation after surgery (Zhang et al., 2016). As shown in **Figures 1B–D**, the inhibition of GA on T24 cells was significantly higher than 5-FU at the same concentration, especially for 24 and 48 h. These results revealed that GA possessed a potent inhibition ability on T24 cells viability at a very low concentration. In view of the possible cytotoxicity caused by excessive drug concentration, the action concentrations of GA were determined to be 6.25, 12.5, and 25 µg/ml, and the action time was determined to be 24 h. In addition, we found that, GA affected T24 cells morphology (**Figure 1E**), significantly inhibited cells proliferation (**Figures 2A, B**), blocked T24 cells cycle in S phase (**Figure 2C**), and induced cell apoptosis, mainly in early stage (**Figure 3A**). Therefore, the subsequent research focused on the molecular mechanism of GA promoting T24 cells apoptosis.

Several researches showed that mitochondria can maintain cell energy supply and its dysfunction will promote apoptosis, which is associated with ROS accumulation and MMP depolarization (Song et al., 2016; Burke, 2017; Wen et al., 2019). Besides, it has been confirmed that ROS generation and MMP depolarization would change intracellular environment and cause oxidative damage even cell apoptosis (Zhang et al., 2015). Our study revealed that mitochondrial ROS level was significantly increased and MMP level was significantly decreased in T24 cells after GA stimulation, which was in accordance with the expectation (**Figures 3B, C**). In previous literature, GA induced tumor cells apoptosis is closely associated with mitochondria dependent endogenous apoptosis pathway (Wang et al., 2016; Gu et al., 2018), so we decided to detect the expression of mitochondrial apoptosis pathway related markers (Bcl-2, Bax, P53, Caspase-3, and Cyt-c). P53 can up-regulate the expression of pro-apoptotic protein Bax, and down-regulate the expression of anti-apoptotic protein Bcl-2, and trigger apoptosis *via* mitochondrial pathway (Bi et al., 2016). Caspase-3 is also called death protease, which can cause chromatin shrinkage, DNA fragmentation, cell lysis, and apoptosis. Several literature have confirmed that Cyt-c could amplify apoptosis signaling and directly regulate apoptosis (Min et al., 2018). Our research showed that, after GA treatment, the proteins and genes expression level of Bax, P53, Caspase-3, and Cyt-c were significantly increased, while Bcl-2 was significantly decreased (**Figures 4** and **7**). NAC pretreatment reversed GA-caused T24 cells apoptosis and MMP level decline as well as Cyt-c secretion from mitochondria to cytoplasm (**Figure 5**). The change trends of these markers were in consistent with the mitochondrial apoptosis pathway reported in relevant literature, suggesting GA induced T24 cells apoptosis may be associated with the mitochondrial dysfunction. Moreover, Ho et al. (Ho et al., 2010) found that GA inhibited gastric cancer AGS cells metastasis *via* NF-κB inhibition and PI3K/Akt/small GTPase signals down-regulation. PI3K/Akt/NF-κB signaling transduction promotes tumor cell growth and metastasis, inhibits tumor cell apoptosis (Roy et al., 2019). Therefore, inhibiting this pathway might be a valid approach for treating various types of cancers. Modern studies verified that some Chinese herbal extracts can promote multiple tumor cells apoptosis *via* PI3K/Akt/NF-κB inhibition (Li et al., 2019; Roy et al., 2019). In this signaling pathway, PI3K firstly promotes Akt phosphorylation to activate or suppress its downstream targets such as Bad, Caspase-9, and NF-κB. Increasing evidences suggested that P-Akt could promote cell survival by regulating the activity of IKKα (the IκB kinase), and promoting IκBα (an inhibitor of NF-κB) phosphorylation (Martini et al., 2014). Finally, the P-IκBα can release protease and NF-κB dimer, enabling NF-κB enter into cell nucleus and activate transcription of the corresponding genes. Our results revealed the relative expression of P-PI3K, P-Akt, P-NF-κB p65, P-IκBα, and P-IKKα proteins were significantly inhibited (**Figure 6**), meanwhile, the relative expression of PI3K, Akt, and NF-κB p65 genes were also significantly inhibited in T24 cells after GA stimulation (**Figure 7**). These results indicated that GA induced T24 cells apoptosis might be closely associated with the inhibition of PI3K/Akt/NF-κB signaling pathway.

In addition, malignant tumors have a high mortality, about 70–80% of cancer patients die for the spread and metastasis of tumor cells. Previous studies reported that GA had anti-metastasis effect *in vitro* and *in vivo* (Ho et al., 2010; Lo et al., 2011). As we all know, bladder cancer is a common malignant tumor of urinary system, it has strong invasive ability and may recur and metastasize after surgery. Therefore, we selected human bladder transitional cell carcinoma T24 cell line for its high infiltration and metastasis activity. Tumor lethality is closely associated with cells migration and invasion, and new vessel formation is the basis of tumor cells growth and metastasis (Ramjiawan et al., 2017). More importantly, VEGF is a main stimulating factor of tumor angiogenesis, it can promote vascular endothelial cells growth and induce vascular proliferation, which is closely associated with the progression of cancers. The relevant studies showed that VEGF is closely related to metastatic activity, and it can induce angiogenesis and promote cancer cells metastasis (Siveen et al., 2017; Melincovici et al., 2018). At present, VEGF inhibitor is an important approach for tumor therapy, and has become an important aspect of targeted therapy. For example, Bevacizumab is the first approved anti-angiogenesis drug in USA, it can be used to treat many different types of tumors and is widely used in clinical practice. In this research, the wound healing assay and transwell migration assay were used to investigate the effects of GA on T24 cells migration. Moreover, the transwell invasion assay was performed to assess the invasion ability of T24 cells. The results indicated that GA significantly inhibited migration and invasion of T24 cells, along with decreased intracellular VEGF protein expression and decreased extracellular VEGF secretion (**Figure 8**).

**Figure 9:**
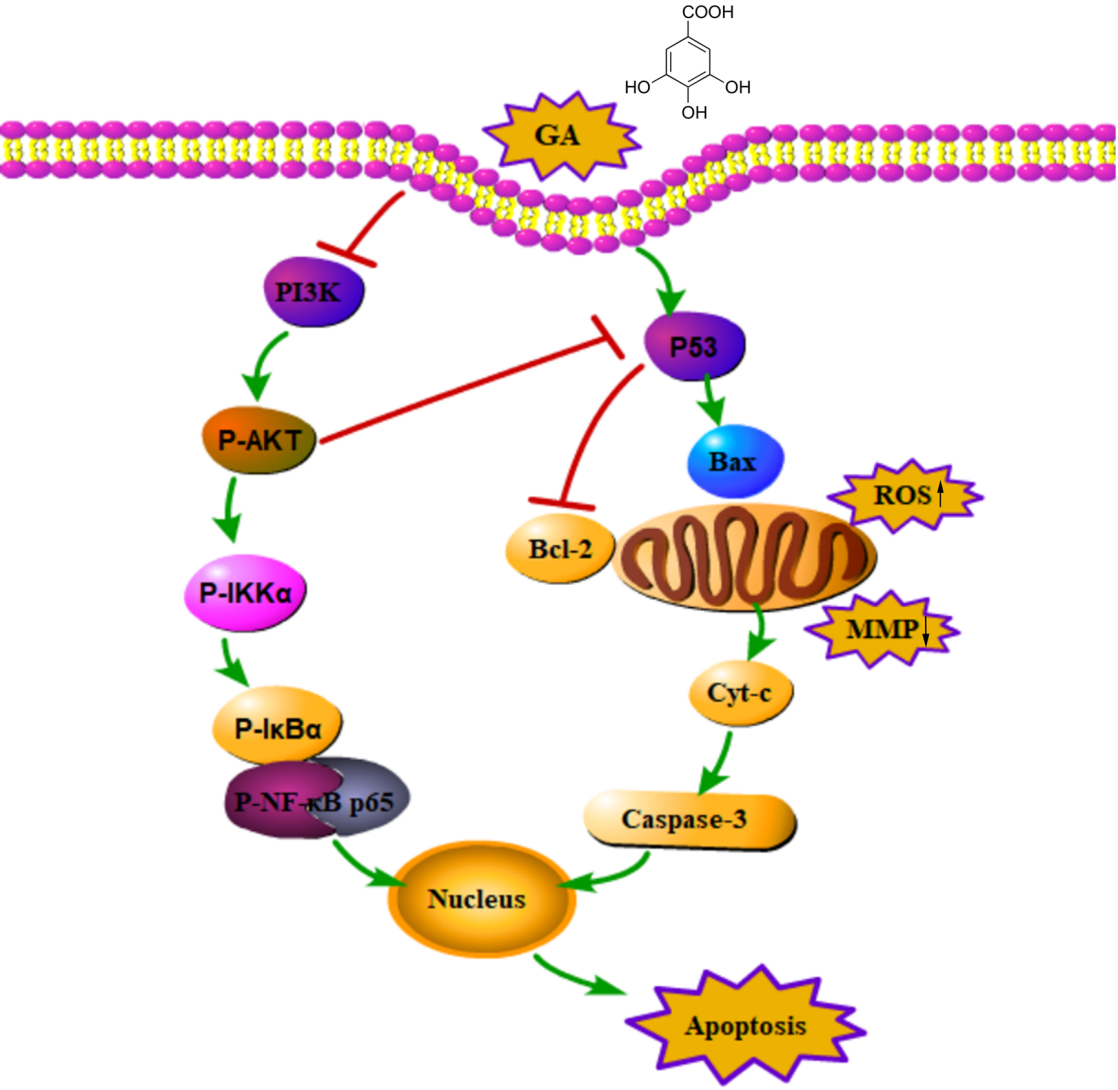
An action mechanism diagram of GA inducing T24 cells apoptosis *via* mitochondrial dysfunction and PI3K/Akt/NF-κB signaling suppression. The → presented for activation, the ┴ presented for inhibition.

In summary, GA inhibited T24 cells proliferation and blocked T24 cells cycle in S phase, moreover, GA significantly induced T24 cells apoptosis, which was closely associated with ROS accumulation and MMP depolarization. The pro-apoptotic effects of GA on T24 cells may be associated with PI3K/Akt/NF-κB signaling pathway. In addition, GA significantly decreased VEGF protein expression and inhibited T24 cells migration and invasion. Taking together, our research demonstrated that GA significantly inhibited T24 cells proliferation and metastasis, and promoted apoptosis, and its pro-apoptotic activity may be closely associated with mitochondrial dysfunction and PI3K/Akt/NF-κB suppression (**Figure 9**). Our findings may provide a safe and effective treatment for bladder cancer. However, the deeper molecular mechanism involved in bladder cancer progression still need further exploration and discussion.

## Data Availability Statement

The raw data supporting the conclusions of this article will be made available by the authors, without undue reservation, to any qualified researcher.

## Author Contributions

MZ and YS led study design and prepared the manuscript. MZ conducted most of the experiments. DJ and YL prepared the reagents and chemicals. KL and QL performed statistical analysis. BZ performed data analysis and interpretation.

## funding

The present study was supported by National Natural Science Foundation of China (No.31570349). The authors are very grateful to the Central Laboratory of Renmin Hospital of Wuhan University for providing technical assistance, experimental places and equipment facilities.

## Conflict of Interest

The authors declare that the research was conducted in the absence of any commercial or financial relationships that could be construed as a potential conflict of interest.
